# Various Forms of Programmed Cell Death Are Concurrently Activated in the Population of Retinal Ganglion Cells after Ischemia and Reperfusion

**DOI:** 10.3390/ijms24129892

**Published:** 2023-06-08

**Authors:** Galina Dvoriantchikova, Emily Adis, Karin Lypka, Dmitry Ivanov

**Affiliations:** 1 Bascom Palmer Eye Institute, Department of Ophthalmology, University of Miami Miller School of Medicine, Miami, FL 33136, USA; 2 Department of Microbiology and Immunology, University of Miami Miller School of Medicine, Miami, FL 33136, USA

**Keywords:** retinal ischemia–reperfusion, retinal ganglion cells, RNA-seq analysis, apoptosis, regulated necrosis, necroptosis, oxytosis/ferroptosis, ferrous iron, deferiprone, death receptors

## Abstract

Retinal ischemia–reperfusion (IR)—which ultimately results in retinal ganglion cell (RGC) death—is a common cause of visual impairment and blindness worldwide. IR results in various types of programmed cell death (PCD), which are of particular importance since they can be prevented by inhibiting the activity of their corresponding signaling cascades. To study the PCD pathways in ischemic RGCs, we used a mouse model of retinal IR and a variety of approaches including RNA-seq analysis, knockout animals, and animals treated with an iron chelator. In our RNA-seq analysis, we utilized RGCs isolated from retinas 24 h after IR. In ischemic RGCs, we found increased expression of many genes that regulate apoptosis, necroptosis, pyroptosis, oxytosis/ferroptosis, and parthanatos. Our data indicate that genetic ablation of death receptors protects RGCs from IR. We showed that the signaling cascades regulating ferrous iron (Fe^2+^) metabolism undergo significant changes in ischemic RGCs, leading to retinal damage after IR. This data suggests that the activation of death receptors and increased Fe^2+^ production in ischemic RGCs promote the simultaneous activation of apoptosis, necroptosis, pyroptosis, oxytosis/ferroptosis, and parthanatos pathways. Thus, a therapy is needed that concurrently regulates the activity of the multiple PCD pathways to reduce RGC death after IR.

## 1. Introduction

Retinal ischemia–reperfusion (IR) is a common mechanism in numerous ocular disorders (e.g., glaucoma, diabetic retinopathy, ischemic optic neuropathy, etc.), the ultimate consequence of which is retinal ganglion cell (RGC) death, leading to visual impairment or even blindness [1,2,3,4]. Currently, our ability to regulate retinal IR pathogenesis remains roughly limited to slowing the rate of degenerative change. The development of novel therapies to halt or reverse retinal degeneration will require a far deeper understanding of the signaling cascades involved in IR-induced RGC death.

It has been shown that IR injury to retinal tissue leads to RGC death by apoptosis and necrosis [5,6,7]. While cell apoptosis is regulated by signaling cascades, cell death by necrosis can be either accidental or regulated via signaling cascades [8,9]. Initially, it was assumed that RGC death by necrosis after retinal IR is not regulated and thus that this form of cell death has no therapeutic value. However, a growing body of evidence suggests that this point of view is wrong. There are numerous types of regulated necrosis including necroptosis, pyroptosis, oxytosis/ferroptosis, and parthanatos, the roles of which have already been shown in retinal IR [6,8,9,10,11,12,13,14,15,16,17,18]. However, the inhibition of either apoptosis or each of these types of regulated necrosis alone did not result in 100% RGC survival after retinal IR [6,12,13,14,15,16,17,18]. In this regard, a recent study by Qin et al. is so important, indicating that several types of programmed cell death (PCD)—apoptosis, necroptosis, and oxytosis/ferroptosis—are activated simultaneously in the retina after IR; concurrent inhibition of these types of PCD led to a significant survival of ischemic RGCs compared to when each type of PCD was inhibited separately [6]. Our recent study indicates that many types of regulated necrosis, including necroptosis, pyroptosis, oxytosis/ferroptosis, and parthanatos, are concurrently triggered in the retina after IR [18]. The disadvantage of these studies was that they were carried out on the entire retina and thus it was difficult to unequivocally state that all these PCD pathways were simultaneously activated in the RGC population and not in other retinal cell types.

The use of high-throughput next-generation sequencing (NGS) technology to study gene expression (RNA-seq analysis) opens up wide opportunities for identifying the signaling cascades involved in IR-induced RGC death. However, the study of RGC transcriptomes using ischemic and control retinas is complicated because these neurons represent less than 5% of the total cell population in this tissue, which may lead to a misinterpretation of the obtained data. To enrich the content of RGCs in a suspension of retinal cells, several methods can be used: the two-step immunopanning protocol, fluorescence-activated cell sorting (FACS), and laser capture microdissection (LCM). However, FACS (due to the low number of RGCs, high level of photoreceptor contamination, and high level of dead cells, resulting in low RNA quantity/quality) and LCM (due to the low number of RGCs and low RNA quantity/quality; other cells can contaminate the sample too) do not provide the required quality/quantity of material necessary for the high-quality RNA-seq analysis. Meanwhile, the two-step immunopanning protocol allows us to isolate a high number of RGCs with high RNA quantity/quality and low contamination by other retinal cells. In our study, for the first time, changes in gene expression were investigated in ischemic vs. control retinal cells in which the proportion of RGCs reached 90%. The high content of RGC transcripts in our RNA-seq data allows us to more unambiguously assert that multiple PCD pathways are simultaneously active in the population of RGCs after IR.

## 2. Results

### 2.1. High-Throughput Gene Expression Analysis Revealed Significant Changes in the Biological Processes Occurring in Ischemic vs. Control RGCs

**Figure 1:**
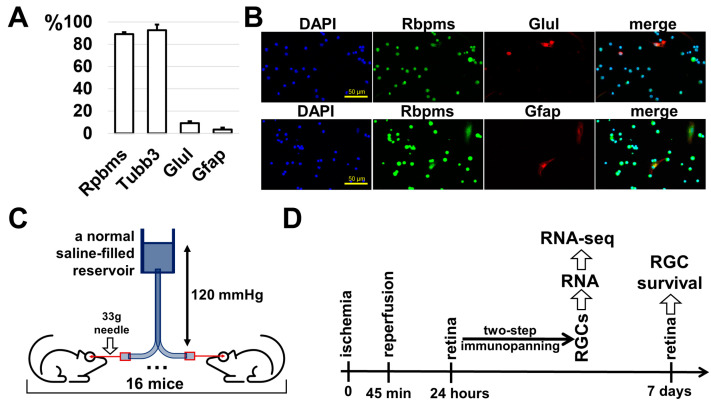
Two-step immunopanning protocol for the collection of highly RGC-enriched samples from retinas of 2-month-old mice that can be used in RNA-seq analysis. (**A**) Percentage of RGCs (Rpbms and Tubb3), Muller glia (Glul), and astrocytes (Gfap) estimated in the cell population isolated from retinas of 2-month-old animals via immunopanning protocol. (**B**) Representative confocal images indicating increased content of RGCs (Rpbms) in the retinal cell population isolated via immunopanning protocol. (**C**) Process of inducing transient retinal ischemia on 16 animals to obtain required number of ischemic and control retinas. In total, we had to perform forty-eight timed surgeries to obtain three biological replicates. (**D**) Ischemic and control retinas collected 24 h after reperfusion used for RGC purification, followed by RNA-seq analysis. Retinas collected 7 days after reperfusion were used to study RGC survival.

Over the past 35 years, the two-step immunopanning protocol has become the leading method for obtaining a viable and highly pure population of RGCs [19]. Our experience indicates that the purity of RGCs obtained from postnatal day (P) 10–12 mouse retinas using this protocol is as high as 95–98% [12,20,21]. This percentage is reduced if adult animals (1 month or older) are used. Since we planned to use 2-month-old animals in our study, we first determined the percentage of RGCs in the population of retinal cells isolated via the immunopanning protocol. To this end, we isolated RGCs from the retinas of 2-month-old mice and cultured them for 24 h so they could attach to coverslips placed in a 24-well cell culture plate. RGCs isolated from adult mice die rapidly in culture and rarely grow neurites. Therefore, we cultured these neurons for only 24 h. Then the cells were fixed, and the cell types/percentages were estimated via immunocytochemistry using cell type specific markers (Rbpms and Tubb3 as RGC markers, Glul as a Muller glia marker, and Gfap as an astrocyte marker). We used DAPI, which stains nuclei, to count the total number of cells. We found that the percentage of Rbpms-positive cells was 89 ± 2%, that of Tubb3-positive cells was 93 ± 5%, that of Glul-positive cells was 9 ± 2%, and that of Gfap-positive cells was 4 ± 2%; *n* = 20, Figure 1A,B). Thus, the percentage of isolated RGCs from the retinas of adult 2-month-old animals was less than that from P10–P12 pups. However, it reached 90%, which is much better than the 5% or less observed in the retina. We also found a high content of glial cells (Muller glia and astrocytes, Figure 1A,B). We can explain this with the fact that neurons form stronger contacts with these glial cells as they age. Thus, treatment of the retinal cell suspension with the papain enzyme (refer to the Section 4) is less efficient for adult animals, which leads to glial cells retaining contact and, together with RGCs, being pulled out of the cell suspension. The higher percentage of Tubb3-positive cells can be explained by the presence of retinal neurons such as photoreceptors, since about 70% of all cells in the adult retina are photoreceptors (anti-Tubb3 antibodies can stain various types of neurons, but they stain RGCs best). However, all these circumstances do not change the fact that the use of the two-step immunopanning protocol makes it possible to significantly enrich the content of RGCs, which is sufficient to study changes in these neurons resulting from ischemia and reperfusion in vivo.

**Figure 2:**
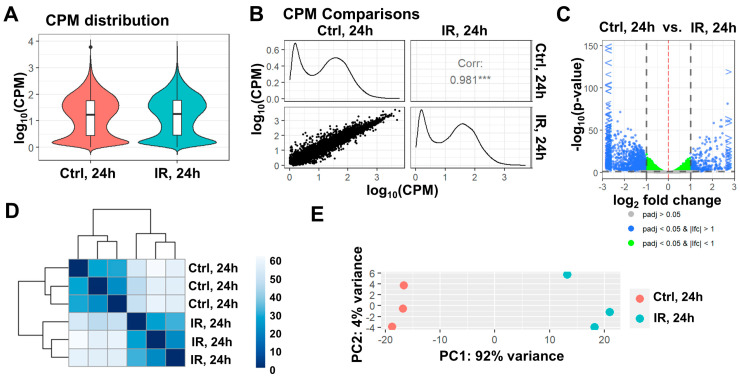
Ischemia and reperfusion significantly change the expression of genes in RGCs. (**A**) Results of the RNA-seq analysis showing that counts per million (CPM) distributions for control (Ctrl) and ischemic (IR) RGCs do not differ significantly 24 h after reperfusion. (**B**) Scatter plot matrix utilized to visualize the correlation (Corr) between gene expression in control (Ctrl) and ischemic (IR) RGCs 24 h after reperfusion. CPM distributions (histograms) and Corr values were generated using the ViDGER Bioconductor package. (**C**) Volcano plot showing that the expression of many genes in ischemic RGCs is statistically significantly changed. Blue and green nodes on the graph represent statistically significant changes in gene expression (padj is *p* value adjusted; lfc is the log2 fold change (LogFC) between two conditions (Ctrl vs. IR)). (**D**,**E**) Sample clustering [a heatmap of sample-to-sample distances (**D**) and principal component analysis (PCA) (**E**)] showing a significant difference between control (Ctrl) and ischemic (IR) RGCs 24 h after reperfusion.

**Figure 3:**
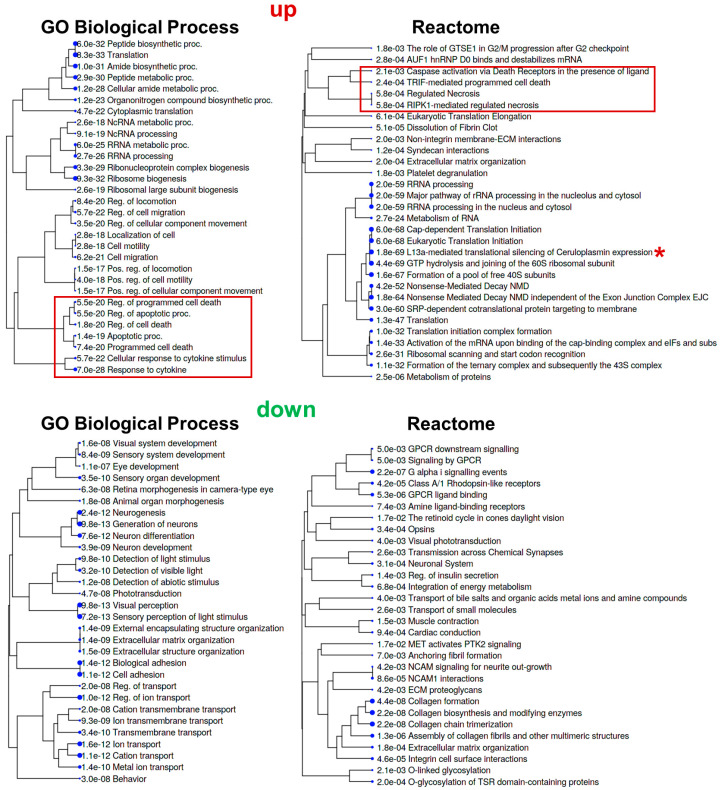
Gene ontology (GO) and pathway analyses revealing that many genes with increased expression in ischemic RGCs are involved in protein biosynthesis and PCD (highlighted in red), while many genes with reduced expression in ischemic RGCs are involved in visual perception and neuronal homeostasis.

The transient retinal ischemia animal model was developed many years ago and perfectly emulates the events that occur during retinal ischemia and reperfusion: the rapid development of an inflammatory response (within the first few hours after reperfusion) and significant RGC death through apoptosis and necrosis [1,5,7,18]. In the first two days after reperfusion, there is a significant predominance of necrosis over apoptosis in the ischemic retina [5,7]. The peak of cell death is observed in the first 24 h after reperfusion. On the third to fourth day after reperfusion, the number of apoptotic cells prevails over necrotic cells. The total number of dead cells decreases significantly by the fourth day, and on the seventh day, dead cells are no longer observed in the ischemic retina. The inflammatory response in the ischemic retina is consistent with the prevalence of necrotic cells over apoptotic cells [18]. However, there were few data available regarding the processes occurring directly in RGCs after retinal IR. To study changes in gene expression in ischemic vs. control RGCs, we simultaneously performed transient retinal ischemia on 16 animals (there was a gap of 2 h between the first and the last animal; Figure 1C). After 24 h, we collected 16 ischemic and 16 control retinas to isolate RGCs from them using the two-step immunopanning protocol (Figure 1D). We used 16 retinas as this allowed us to isolate 700,000–900,000 total RGCs, which corresponds to about 1000 nanograms of high-quality RNA. We repeated this experiment to retrieve three biological replicates, which amounted to forty-eight timed surgeries in total to obtain three biological replicates. Ischemic and control RGCs were used for the preparation of RNA-seq libraries for NGS (three samples of ischemic RGCs and three samples of control RGCs, amounting to six RNA-seq libraries total). While 54,319,921 ± 2,178,237 fragments (or more than 100 M reads) on average per library were sequenced, 43,468,817 ± 1,892,615 fragments were uniquely mapped to the mouse genome. To perform the differential expression analysis of our RNA-seq data, we used the edgeR Bioconductor package [22]. While counts per million (CPM) distributions for ischemic and control RGCs are relatively similar, our data indicate that gene expression in ischemic RGCs is significantly altered compared to that in control RGCs; the expression of 7335 genes changed at 24 h (FDR < 0.1, Figure 2A, Supplementary Data, S1). This difference is clearly seen from the volcano plot, sample clustering, and principal component analysis (PCA) (Figure 2). Next, we selected genes whose expression was statistically significantly (FDR < 0.1) increased or decreased by one and a half times in ischemic RGCs compared to that in control ones (logFC > 0.585 or logFC < −0.585). We used lists of these genes in the gene ontology (GO) and pathway analyses (the GO knowledgebase and Reactome pathway database; Figure 3, Supplementary Data, S1). We found that many of the upregulated genes are associated with protein biosynthesis (e.g., GO translation (enrichment FDR = 8.3 × 10^−33^), GO peptide biosynthetic proc. (6 × 10^−32^), GO peptide metabolic proc. (2.9 × 10^−30^), GO ribosome biogenesis (9.3 × 10^−32^), Reactome translation (1.3 × 10^−47^), Reactome translation initiation complex formation (1.0 × 10^−32^), etc.; Figure 3). Our data also indicate that the expression of many genes involved in programmed cell death (PCD) is increased (e.g., GO Reg. of cell death (enrichment FDR = 1.8 × 10^−20^), GO programmed cell death (7.4 × 10^−20^), GO apoptotic proc. [1.4 × 10^−19^], GO reg. of programmed cell death (5.5 × 10^−20^), Reactome TRIF-mediated programmed cell death (2.4 × 10^−4^), Reactome RIPK1-mediated regulated necrosis (5.8 × 10^−4^), Reactome regulated necrosis (5.8 × 10^−4^), etc.; highlighted in red, Figure 3). Meanwhile, the GO and pathway analyses revealed that the expression of genes associated with the visual perception and neuronal homeostasis was reduced (e.g., GO visual perception (enrichment FDR = 9.8 × 10^−13^), GO Generation of neurons (9.8 × 10^−13^), GO reg. of ion transport (1.0 × 10^−12^), GO ion transport (1.6 × 10^−12^), Reactome neuronal system (3.1 × 10^−4^), Reactome NCAN signaling for neurite out-growth [4.2 × 10^−3^], Reactome transmission across chemical synapses (2.6 × 10^−3^), etc.; Figure 3).

### 2.2. The Signaling Cascades That Regulate Ferrous Iron (Fe^2+^) Metabolism Undergo Significant Changes in Ischemic RGCs

**Figure 4:**
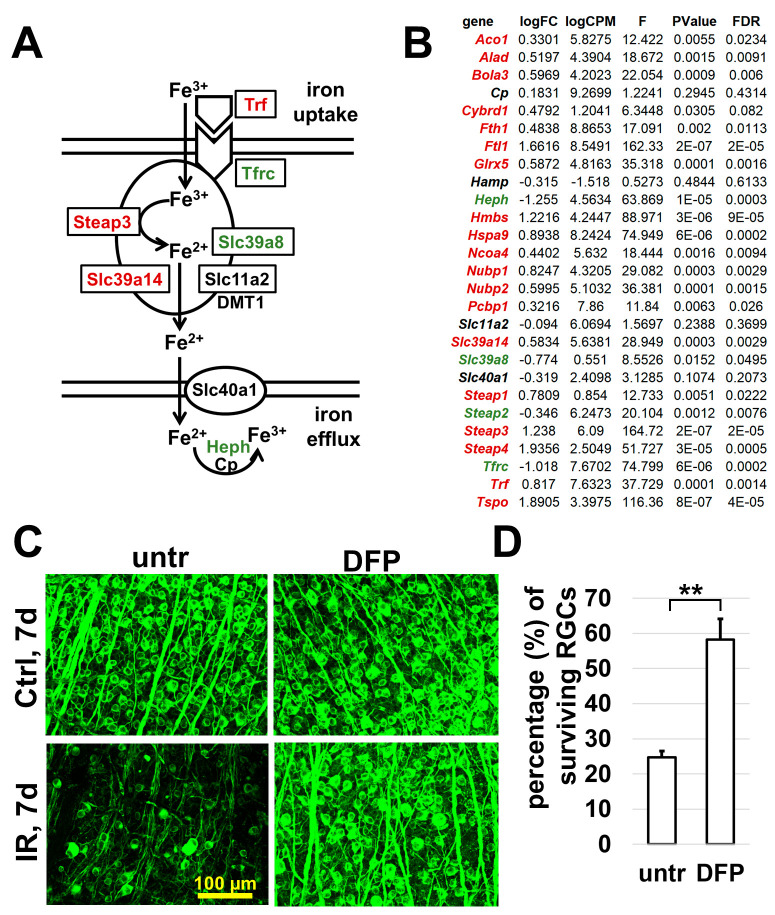
Ischemia–reperfusion leads to significant changes in iron metabolism, which affects RGC survival. (**A**) Changes in expression of genes involved in iron metabolism suggesting that ferrous iron (Fe^2+^) is likely to accumulate in ischemic RGCs. (**B**) Expression of many genes involved in iron metabolism being statistically (FDR < 0.1; highlighted in dark red) significantly increased in ischemic RGCs. The genes whose expression is statistically significantly reduced are highlighted in green. (**C**) Representative confocal images showing that a decrease in iron levels in the ischemic retina leads to increased survival of RGCs 7 days after reperfusion. (**D**) Graph showing the percentage of surviving RGCs in ischemic retinas of DFP treated and control (untr) animals 7 days after reperfusion (** *p* value  <  0.001).

As a result of the pathway analysis, we have found that the “Reactome L13a-mediated translational silencing of Ceruloplasmin expression” pathway has the highest enrichment FDR (1.8 × 10^−69^; Figure 3, marked with a red star). Ceruloplasmin (Cp) is one of the proteins that regulate iron metabolism in the cell. Increased expression of this gene has been found in the retinas of humans and animals suffering from glaucoma and in an optic nerve crush model [23,24,25]. However, we did not find significant changes in *Cp* expression in ischemic RGCs compared to controls (logFC = 0.18, FDR = 0.43), which may be associated with the activation of the “Reactome L13a-mediated translational silencing of Ceruloplasmin expression” pathway. These observations have aroused our interest in analyzing the signaling cascades that regulate iron metabolism. We found that the expression of genes, whose activity leads to high ferrous iron (Fe^2+^) production in cells, was significantly increased in ischemic RGCs compared to that in the control RGCs (Figure 4A,B, Supplementary Data, S2). Meanwhile, the expression of genes that promote the release of iron from the cell and its conversion from the ferrous (Fe^2+^) into the ferric (Fe^3+^) form was reduced or unchanged (Figure 4A,B, Supplementary Data, S2). All these processes may contribute to the accumulation of ferrous iron (Fe^2+^) in ischemic RGCs. In its free form (as a catalyst of the Fenton/Haber–Weiss reaction), ferrous iron (Fe^2+^) leads to the production of a huge amount of reactive oxygen species (ROS), resulting in oxidative stress and cell death [26,27]. In this regard, it is worth noting the *Steap3* gene encoding the enzyme responsible for the generation of ferrous (Fe^2+^) iron from ferric (Fe^3+^) iron [28]. The expression of *Steap3* was more than twice as high in ischemic RGCs compared to that in control RGCs (logFC = 1.24, FDR = 1.5 × 10^−5^). Of note is that the expression of two other members of this family was also upregulated in ischemic RGCs (*Steap1*: logFC = 0.78, FDR = 0.02; *Steap4*: logFC = 1.94, FDR = 5 × 10^−4^. Figure 4B, Supplementary Data, S2). Thus, a high expression of the STEAP family enzymes in ischemic vs. that in control RGCs may lead to the generation of large amounts of free ferrous (Fe^2+^) iron, which may have a dramatic impact on the wellbeing of ischemic RGCs.

To evaluate the significance of IR-induced changes in iron metabolism, we treated ischemic mice using the oral iron chelator deferiprone (DFP). DFP significantly lowers labile iron levels and oxidative stress even after short-term use, protecting the retina from many pathological conditions [29,30,31,32,33]. To this end, one group (DFP) of mice received oral DFP (1 mg/mL in drinking water) within 8 days before IR, while the other did not (serving as a control (untr)). Retinal IR was then induced in both groups of animals. Group “DFP” continued to receive the drug for the next 7 days after IR, while group “untr” did not. To quantify the number of surviving RGCs, the retinas of these animals were collected 7 days after IR, and whole retina flat mounts were stained using the RGC marker Tubb3 (Figure 4C). We found that the number of viable RGCs was significantly higher in DFP–treated retinas compared to that in controls (58 ± 6% vs. 24 ± 2%, *p* value < 0.001, *n* = 5) (Figure 4D). These data provide evidence of the critical role that iron metabolism plays in retinal damage and RGC death after IR. Since iron plays a key role in oxytosis/ferroptosis, these data also indicate an important role of oxytosis/ferroptosis in retinal IR.

### 2.3. Ischemia–Reperfusion Activate Simultaneously a Variety of Signaling Cascades That Regulate Many Types of PCD in RGCs

The increased expression of genes that regulate or are directly involved in PCD (GO reg. of cell death, GO programmed cell death, GO apoptotic proc., GO reg. of programmed cell death, Reactome TRIF-mediated programmed cell death, Reactome RIPK1-mediated regulated necrosis, and Reactome-regulated necrosis; highlighted in red, Figure 3) prompted us to study the corresponding signaling cascades in more detail. To this end, we have compiled a list of genes involved in apoptosis and regulated necrosis (Supplementary Data, S3). We found that the expression of many apoptosis triggering genes was significantly upregulated in ischemic RGCs (e.g., *Bid*, BIM (*Bcl2l11*), *Bak1*, *Hrk*, *Casp8*, *Ddit3*, etc.; Table 1, Supplementary Data, S3). The expression of many genes involved in regulated necrosis was also statistically significantly increased in ischemic vs. that in control RGCs; these involved genes of necroptosis (*Ripk1*, *Ripk3*, and *Mlkl*), pyroptosis (e.g., ASC (*Pycard*), *Zbp1*, and *Casp1*), oxytosis/ferroptosis (e.g., *Acsl5*, *Ftl1*, *Hmox1*, *Lpcat3*, *Slc39a14*, *Steap3*, *Trf*, *Trp53*, etc.), and parthanatos (*Parp1*). It has been previously shown that all these types of PCD (apoptosis, necroptosis, pyroptosis, oxytosis/ferroptosis, and parthanatos) are individually responsible for the death of RGCs after IR [6,12,13,14,15,16,17,18]. Since iron metabolism plays a key role in oxytosis/ferroptosis, our present study confirms the important role of oxytosis/ferroptosis in RGC death after retinal IR (Figure 4). However, all these data also indicate that the expression of genes that promote apoptosis and regulated necrosis is increased simultaneously in the population of RGCs after IR (Table 1, Supplementary Data, S3). Thus, we can conclude that signaling cascades that regulate or are directly involved in apoptosis, necroptosis, pyroptosis, oxytosis/ferroptosis, and parthanatos are triggered simultaneously in the population of ischemic RGCs and thus deserve attention not individually but collectively.

**Figure 5:**
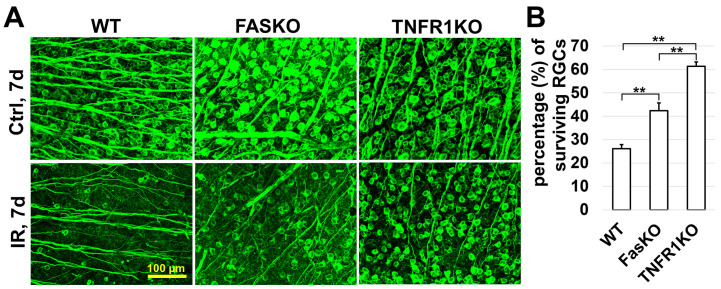
The activity of death receptors significantly contributes to RGC death after retinal IR. (**A**) Representative confocal images showing that genetic ablation of TNFR1 and FAS protects RGCs from IR. (**B**) Graph showing percentage of surviving RGCs in ischemic retinas of WT mice and mice with reduced activity of death receptors 7 days after reperfusion (** *p* value < 0.001).

In addition to the genes directly involved in PCD, we found an increase in expression of many receptors and their second messengers, the activation of which can lead to apoptosis and regulated necrosis. We found increased expression of toll-like receptor 4 (TLR4/*Tlr4*), TRIF (*Ticam1*), and *Myd88*, whose activities lead to significant RGC death after retinal IR (Table 1, Supplementary Data, S1) [34,35]. Our data also indicate an increased expression of many death receptors such as TNFR1 (*Tnfrsf1a*), TRAILR (*Tnfrsf10b*), and FAS (*Fas*) (Table 1). These receptors are activated by cytokines such as TNF, TRAIL, and FASL. These results are consistent with the statistically significant enrichment FDRs observed by us in the GO and pathway analysis (GO cellular response to cytokine stimulus (5.7 × 10^−22^), GO response to cytokine (7.0 × 10^−28^], and Reactome caspase activation via death receptors in the presence of the ligand (2.1 × 10^−3^), Figure 3). The role of TNFR1 (*Tnfrsf1a*) in RGC death after retinal IR has already been shown [18,36]. However, the role of other death receptors in RGC death after retinal IR has not yet been investigated. Here, we examined the contribution of FAS (*Fas*) to RGC death after retinal IR and compared its contribution to that of TNFR1 (*Tnfrsf1a*). To this end, retinal ischemia was induced in TNFR1 and FAS knockout animals (TNFR1KO and FASKO). The retinas of these animals were collected 7 days after reperfusion and were stained for the RGC marker Tubb3 to quantify the number of surviving RGCs (Figure 5A). We found that while genetic ablation of TNFR1 and FAS protects RGCs from retinal IR, TNFR1 plays a more significant role in retinal IR than FAS does because the deletion of TNFR1 results in greater protection (TNFR1KO: 61 ± 2%, FASKO: 42 ± 3%, WT: 26 ± 2%, *p* value < 0.001, *n* = 5; Figure 5B). However, this does not diminish the importance of FAS (*Fas*) and other death receptors in retinal IR.

## 3. Discussion

Whether chronic or acute, retinal IR has been implicated in a myriad of retinal disorders, including ischemic optic neuropathy, glaucoma, and diabetic retinopathy [1,2,3,4]. Retinal IR ultimately leads to RGC death by apoptosis, accidental necrosis, and regulated necrosis [5,6,7,12,13,14,15,16,17,18]. Special attention should be paid to various types of programmed cell death (PCD), since they can be prevented by inhibiting the activity of their corresponding signaling cascades [8,9]. Our data indicate that many of these signaling cascades are triggered simultaneously in the population of ischemic RGCs 24 h after reperfusion. The activation of death receptors and toll-like receptors on the surface of ischemic RGCs, and increased ferrous iron (Fe^2+^) production in these neurons may be responsible for the simultaneous triggering of many of these PCD pathways. These observations should be taken into account to create highly efficient drugs for the treatment of retinal IR.

Apoptosis of RGCs was one of the first types of PCD shown in the retina after IR [5,6,7]. RGC death by necroptosis, pyroptosis, oxytosis/ferroptosis, and parthanatos after IR was shown later [6,12,13,14,15,16,17,18]. The big difference between apoptosis compared to regulated necrosis is that apoptotic cells keep the plasma membrane intact while in regulated necrosis the membrane integrity is compromised [8,9]. From an immunological perspective, apoptosis is an anti-inflammatory form of cell death, while the release of endogenous factors (proteins, RNA, DNA, etc., known as damage-associated molecular patterns or DAMPs) from cells that have lost membrane integrity triggers a strong cytotoxic pro-inflammatory response such as in the case of necrosis [37,38,39,40,41,42,43]. It has been shown that the released DAMPs cause inflammation and retinal damage after IR [7,12,13,34,35,44,45,46]. Therefore, necrotic PCD can trigger further RGC death after IR (secondary injury). An increase in the number of apoptotic cells would make it possible to slow down this process by lowering the neurotoxic pro-inflammatory response [47]. Thus, since all these PCD pathways are activated simultaneously in the population of ischemic RGCs, cross-talk between apoptotic and necrotic PCD pathways may determine the final level of damage in the retina after IR.

**Figure 6:**
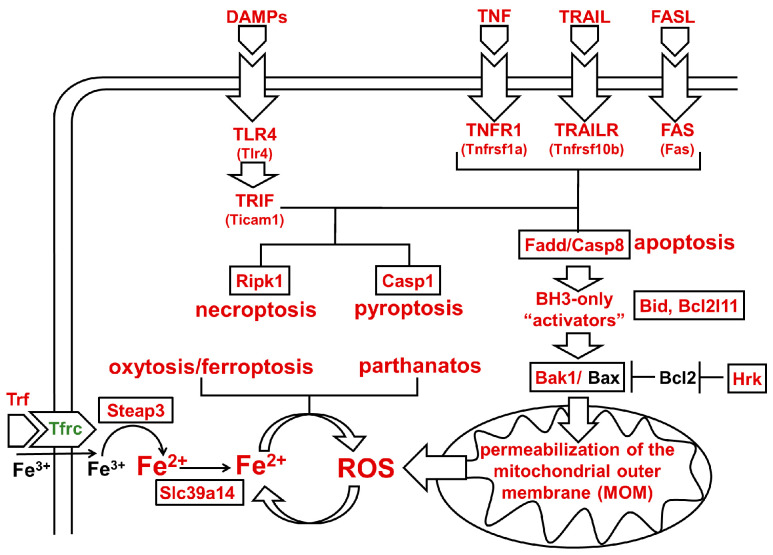
Activation of death and toll-like receptors, and accumulation of ferrous (Fe2+) iron may concurrently trigger apoptosis, necroptosis, pyroptosis, oxytosis/ferroptosis, and parthanatos signaling cascades in the population of ischemic RGCs.

The findings from this study allow us to propose a model that could explain the simultaneous activation of signaling cascades that regulate many types of PCD in the population of ischemic RGCs (Figure 6). Initial ischemic stress leads to accidental (uncontrolled) RGC necrosis (primary injury) and the release of DAMPs from necrotic cells. DAMPs act through toll-like receptor 4 (TLR4/*Tlr4*) located on the surface of glial cells and promote the production of neurotoxic pro-inflammatory factors, including TNF, TRAIL, and FASL [12,34,35,44,46]. Furthermore, TNF, TRAIL, and FASL, acting through RGC death receptors TNFR1 (*Tnfrsf1a*), TRAILR (*Tnfrsf10b*), and FAS (*Fas*), trigger extrinsic apoptosis in some ischemic RGCs via the Fadd/Casp8 signaling cascade (Figure 6). The increased expression of the death receptors in ischemic RGCs makes these neurons more sensitive to the presence of the death receptor ligands in the extracellular space. If an RGC does not die via the extrinsic pathway of apoptosis, then the binding of BH3-only proteins such as Bid (cleaved by Casp8) and BIM (*Bcl2l11*) to Bak1/Bax leads to the permeabilization of the mitochondrial outer membrane. Bcl2 could prevent this process if its activity is not blocked by BH3-only proteins/sensitizers such as Hrk. It has been previously shown that optic nerve transection leads to an increase in the expression of Hrk in RGCs and their subsequent death [48]. Our data indicate that the expression of *Casp8*, *Bid*, BIM (*Bcl2l11*), *Bak1*, and *Hrk* is increased in ischemic RGCs, while *Bcl2* expression is unchanged (Table 1, Supplementary Data, S3). The increased expression of these genes suggests that the permeabilization of the mitochondrial outer membrane is inevitable, and leads to activation of the intrinsic pathway of apoptosis and to the increased production of ROS [49,50]. However, according to our data, the expression of key genes involved in the intrinsic pathway of apoptosis (*Apaf1* and *Casp9*) does not change in ischemic RGCs (Supplementary Data, S3). Therefore, we suggest that the role of extrinsic apoptosis is more significant than the role of intrinsic apoptosis in ischemic RGCs. Meanwhile, the critical role of ROS in IR-induced RGC death and retinal degeneration has already been well documented [1,6,20,51,52,53,54]. In our study, we found an important player that can significantly increase the negative effects of ROS in ischemic RGCs. Our data indicate an important role for ferrous iron (Fe^2+^) in ischemic RGCs (Figure 4). Previously published studies supported this observation [6,17,18]. While individually ROS and ferrous iron (Fe^2+^) do not pose a threat to the cell, their simultaneous presence in the cell are a “thermonuclear mix” that starts the uncontrolled production of ROS from the Fenton/Haber–Weiss reaction, which is controlled by ferrous iron (Fe^2+^) [26]. Huge amounts of ROS can cause significant DNA and membrane damage, leading to RGC parthanatos and/or oxytosis/ferroptosis [8,9,10,11]. We should also note that the death receptors (TNFR1 (*Tnfrsf1a*), TRAILR (*Tnfrsf10b*], and FAS (*Fas*)) and toll-like receptors (TLR4/*Tlr4*) can trigger necroptosis or pyroptosis in ischemic RGCs via their second messengers (*Ripk1*, TRIF (*Ticam1*), *Zbp1*, etc.), whose expression is also upregulated (Table 1 and Figure 6) [8,9]. The important role of death receptor (TNFR1 and FAS) and TLR4/TRIF/Ripk1 signaling cascades in retinal IR has been shown previously as well as in this study (Figure 5) [12,18,34,35,36,44]. Thus, multiple types of PCD can be activated simultaneously in the population of ischemic RGCs within this model (Figure 6). Our model does not assume that all these events occur in the same ischemic RGC. The purpose of this model is to explain how several PCD pathways can be triggered in a population of ischemic RGCs at once.

Finally, through an analysis of gene expression in RGCs isolated from ischemic and control retinas, our experiments with death receptor deficient animals and animals treated with an iron chelator showed that many signaling cascades that regulate multiple types of PCD are simultaneously activated in ischemic RGCs 24 h after reperfusion. Cross-talk between these pathways may determine the final level of RGC death after IR. Thus, a therapy is needed that could concurrently regulate the activity of many PCD pathways to significantly reduce retinal damage after IR [6,17,55,56,57].

## 4. Materials and Methods

### 4.1. Animals and Ethics Statement

All procedures were performed in compliance with the National Institutes of Health (NIH) Guide for the Care and Use of Laboratory Animals and according to the University of Miami Institutional Animal Care and Use Committee (IACUC)’s approved protocol (IACUC 21-070). FAS and TNFR1 knockout animals (FASKO and TNFR1KO; these animals have the C57BL/6 J genetic background), and C57BL/6 J mice as the wild-type (WT) controls were obtained from the Jackson Laboratory (Bar Harbor, ME, USA; stock numbers 000482, 003242, and 000664). One of the groups of WT animals was treated with the oral iron chelator deferiprone (DFP, 1 mg/mL in drinking water; #379409, MilliporeSigma, St. Louis, MO, USA), while the other was not (and served as a control (untr)). The animals were treated with DFP 8 days before retinal ischemia–reperfusion and 7 days after. Animals were given fresh DFP daily in their drinking water to avoid the possibility of its degradation through prolonged exposure to water. Since mice consume 5 mL of fluid per day, each mouse consumed 5 mg of DFP daily. We used 2-month-old mice, which were housed under standard conditions of humidity and temperature, free access to food and water, and a 12 h light to dark cycle. All methods were completed and reported in accordance with ARRIVE guidelines.

### 4.2. Transient Retinal Ischemia

Transient retinal ischemia was induced as described previously [12,35,44]. Briefly, the animals were anesthetized with isoflurane, and ischemia was induced for 45 min by introducing a 33-gauge needle into the anterior chamber of the left eye. The needle was attached to a 0.9% sodium chloride IV injection bag (500 mL) raised 163 cm above the mouse to produce a sodium chloride flow pressure of 120 mmHg (Figure 1C). Complete retinal ischemia was confirmed by the whitening of the anterior segment of the eye and blanching of the retinal arteries via an ophthalmoscope. The right eyes were kept as normotensive controls. Mice were euthanized 24 h or 7 days after reperfusion in accordance with the recommendations of the Panel on Euthanasia of the American Veterinary Medical Association (AVMA). We used 2-month-old male mice in the transient retinal ischemia model since adult female retinas are more resistant to ischemic injury than are adult male retinas [58].

### 4.3. Isolation of Retinal Ganglion Cells (RGCs)

Primary RGCs were isolated according to the two-step immunopanning protocol as described previously [19]. Briefly, the retinas were incubated in papain solution (16.5 U/mL; Worthington Biochemical Corp., Lakewood, NJ, USA) for 30 min to get the single cell suspension. Macrophages and endothelial cells were then removed from this suspension by panning with the anti-macrophage antibody (AIA31240, Accurate Chemical, Carle Place, NY, USA). Next, RGCs were bound to the panning Petri dish containing the antibody against CD90.2/Thy1.2 and were then released by trypsin incubation. The two-step immunopanning protocol takes a few hours to isolate the RGCs, which may affect RNA-seq data. However, previous studies have shown that observed changes are minor, affecting mostly the activity of immediate early genes [59,60,61].

### 4.4. Immunocytochemistry

Isolated primary RGCs were cultured for 24 h and then were fixed in 4% paraformaldehyde (PFA). Next, the cells were blocked in PBS (pH 7.4) buffer containing 5% donkey serum and 0.15% Tween-20. Cells were incubated overnight in PBS/0.2% Triton X-100/10% donkey (or goat) serum buffer containing either anti-Tubb3 antibody (#802001, BioLegend, San Diego, CA, USA), anti-Rbpms antibody (GTX118619, GeneTex, Irvine, CA, USA), Cy3-conjugated anti-Gfap antibody (MAB3402C3, MilliporeSigma, St. Louis, MO, USA), or anti-Glul antibody (anti-Glutamine Synthetase; MAB302; MilliporeSigma, St. Louis, MO, USA), followed by species-specific secondary fluorescent antibodies (ThermoFisher Scientific, Waltham, MA, USA). Negative controls were incubated with secondary antibody only. Imaging was performed using a Leica STELLARIS confocal microscope (Leica Microsystems, Wetzlar, Germany).

### 4.5. RNA Extraction, RNA Quality Control, RNA-seq Library Preparation and Sequencing

Total RNA was purified from isolated RGCs using RNeasy Plus Mini Kit (#74134, Qiagen, Hilden, Germany). The quality and quantity of RNA was assessed using Qubit 4 Fluorometer and the NanoDrop ND-1000 spectrophotometer (ThermoFisher Scientific, Waltham, MA, USA). Briefly, 2100 Bioanalyzer Instrument (Agilent Technologies, Santa Clara, CA, USA) was used to evaluate RNA integrity. The RNA samples, which had a RIN score of 8 or higher, were utilized to prepare RNA-seq libraries using Illumina Stranded mRNA Prep Kit (#20040532, Illumina, San Diego, CA, USA) and IDT^®^ for Illumina^®^ RNA UD Indexes Set A (#20040553, Illumina, San Diego, CA, USA). The quality/quantity of the final RNA-seq libraries were evaluated using Qubit 4 Fluorometer and 2100 Bioanalyzer Instrument. The libraries were multiplexed and then, sequenced on the Illumina Novaseq 6000 with a 2 × 150 paired-end (PE) configuration. The next-generation sequencing (NGS) was performed at the University of Michigan Advanced Genomics Core. The datasets acquired in this study are available in the BioProject database, accession number PRJNA892577.

### 4.6. RNA-seq Data Analysis

A basic STAR workflow was used to align paired-end reads [62]. We used the HTseq package to determine how many sequencing reads overlap each of the mouse genes [63]. The differential gene expression analysis was performed using the edgeR Bioconductor package [22]. The pathway analysis and gene ontology (GO) analysis were performed using ShinyGO 0.76 (http://bioinformatics.sdstate.edu/go/). We utilized ViDGER (visualization of differential gene expression results using R) for visualizations of our data.

### 4.7. Immunohistochemistry and Counting RGCs in the Ganglion Cell Layer

Mouse eyes were fixed with 4% PFA and then were transferred to PBS. The retinas were removed, permeabilized with 0.5% Triton X-100, blocked with 0.5% Triton X-100 containing 10% donkey serum, and then incubated overnight in 0.2% Triton X-100/10% donkey serum in PBS containing the anti-beta III Tubulin antibody (anti-Tubb3; 802,001, BioLegend, USA). The next day, a donkey anti-rabbit secondary antibody (ThermoFisher Scientific, USA) in 0.15 % Tween 20/PBS was applied for 1.5 h at room temperature. After washing with 0.15 % Tween 20/PBS, retinas were flat-mounted (RGC layer facing up), cover-slipped, and imaged with a Leica STELLARIS confocal microscope (Leica Microsystems, USA). Tubb3-positive RGCs were imaged randomly to collect images from four retinal quadrants and were counted with ImageJ software (version 1.53o; https://imagej.nih.gov/; accessed on 11 January 2022 to download ImageJ). RGC survival in the IR retinas was calculated as a percentage of the mean cell density in normotensive fellow control eyes.

### 4.8. Statistical Analysis

The unpaired Student’s *t*-test was employed for experiments containing one variable. *p*-values equal to or less than 0.05 were considered statistically significant. The generation and analysis of NGS data were performed in-house according to ENCODE standards and pipelines with n = 3 for RNA-seq.

## Figures and Tables

**Table 1:** The expression of genes regulating many types of PCD is statistically significantly increased in RGCs of the IR retina 24 h after reperfusion.

Gene	Function	logFC	logCPM	F	*p* Value	FDR
*Tnfrsf1a*	activation of apoptosis, necroptosis	2.277	5.9533	260.28	2 × 10^−8^	6 × 10^−6^
*Tnfrsf10b*		1.9381	1.9099	56.346	2 × 10^−5^	0.0004
*Fas*		1.9164	1.6485	51.171	3 × 10^−5^	0.0005
*Fadd*		0.9508	2.7288	38.059	0.0001	0.0013
*Tlr4*	activation of necroptosis, pyroptosis	1.1634	0.0889	17.894	0.0018	0.0101
*Ticam1*		1.5626	2.9744	133.39	4 × 10^−7^	3 × 10^−5^
*Irf9*		1.2944	5.1371	200.16	6 × 10^−8^	1 × 10^−5^
*Eif2ak2*		1.0321	3.583	25.279	0.0005	0.0042
*Zbp1*		1.8483	−0.349	26.42	0.0004	0.0037
*Bcl2l11*	apoptosis	0.6175	3.5875	22.442	0.0008	0.0058
*Bid*		0.6971	3.5261	36.141	0.0001	0.0015
*Hrk*		2.9852	1.8421	190.93	8 × 10^−8^	1 × 10^−5^
*Bax*		0.5085	4.4379	13.477	0.0043	0.0196
*Bak1*		0.6404	4.3704	34.732	0.0002	0.0017
*Bnip3*		−0.698	7.1945	53.409	3 × 10^−5^	0.0005
*Casp8*		2.532	2.3705	103.56	1 × 10^−6^	6 × 10^−5^
*Casp9*		−0.036	5.3958	0.1677	0.6909	0.7845
*Casp3*		0.4875	4.175	15.429	0.0028	0.0144
*Casp7*		0.2869	1.9767	2.3646	0.1552	0.271
*Ddit3*		0.8363	6.177	64.697	1 × 10^−5^	0.0003
*Capn2*		0.8192	6.5436	42.149	7 × 10^−5^	0.001
*Ripk1*	necroptosis	0.827	3.9544	28.649	0.0003	0.003
*Ripk3*		2.7732	−1.603	12.124	0.0059	0.0247
*Mlkl*		2.1475	−1.25	24.814	0.0006	0.0044
*Pycard*	pyroptosis	0.673	0.4375	7.5547	0.0206	0.0618
*Casp1*		2.1096	−1.467	19.142	0.0014	0.0086
*Gsdma*		2.015	0.3007	39.133	1 × 10−4	0.0012
*Gsdmd*		0.2458	0.7427	0.7417	0.4093	0.545
*Nek7*		0.0218	4.9421	0.0459	0.8346	0.8918
*Acsl3*	oxytosis/ferroptosis	−1.142	8.9786	111.39	1 × 10^−6^	5 × 10^−5^
*Acsl5*		0.8749	5.7817	71.994	7 × 10^−6^	0.0002
*Ftl1*		1.6616	8.5491	162.33	2 × 10^−7^	2 × 10^−5^
*Gpx1*		1.0313	5.5895	119.59	7 × 10^−7^	4 × 10^−5^
*Gpx4*		0.6991	7.9878	64.656	1 × 10^−5^	0.0003
*Gss*		0.6487	3.9966	40.957	8 × 10^−5^	0.0011
*Hmox1*		3.2467	5.534	422.53	2 × 10^−9^	2 × 10^−6^
*Lpcat3*		0.7455	4.1796	28.441	0.0003	0.003
*Sat1*		0.5846	6.9977	7.0402	0.0242	0.0695
*Slc39a14*		0.5834	5.6381	28.949	0.0003	0.0029
*Slc39a8*		−0.774	0.551	8.5526	0.0152	0.0495
*Steap3*		1.238	6.09	164.72	2 × 10^−7^	2 × 10^−5^
*Tfrc*		−1.018	7.6702	74.799	6 × 10^−6^	0.0002
*Trf*		0.817	7.6323	37.729	0.0001	0.0014
*Trp53*		1.024	5.2341	99.392	2 × 10^−6^	7 × 10^−5^
*Aifm1*	parthanatos	0.1282	4.8196	2.0983	0.1782	0.2989
*Parp1*		0.467	6.2068	27.417	0.0004	0.0033
*Mif*		0.2033	7.7412	5.5227	0.0407	0.1011

## Data Availability

The datasets generated and analyzed during the current study are available in the BioProject database (accession number PRJNA892577) and in the article/Supplementary Data.

## Supplementary Materials

The supporting information can be downloaded at https://www.mdpi.com/article/10.3390/ijms24129892/s1.
